# Naphtha in Consumption

**Published:** 1848-06

**Authors:** 


### SELECTIONS

FROM

### AMERICAN AND FOREIGN JOURNALS.

---

*Naphtha in Consumption.*—Dr. THEOPHILUS THOMPSON made some remarks at the meeting of the Medical Society of London, (Dec. 20, 1847,) on the use of naphtha in consumption. He had thought it his duty to ascertain, as far as lay in his power, the effects of this remedy at the Consumptive Hospital With this view, having taken proper precautions to get the agent pure, and to give it as described by its admirers, he administered it in several cases. The conclusions arrived at by these experiments were as follows:—In the third stage of consumption it did positive mischief. In a few cases, when the bronchial tubes were affected, and there was much expectoration, it seemed to arrest the secretion to some extent, lessen the night perspiration, make the pulse fuller and slower, and somewhat relieve the cough; the appetite also improved. In all the stages of consumption, and under other circumstances in the disease, except those mentioned, the naphtha did positive harm. It had no effect whatever on the tubercular disease.

Dr. Golding Bird declared that there never was a greater delusion than to suppose that naphtha was a remedy for consumption. He had employed it, with all the care and caution recommended by its advocates, and he had never found it of service. The fact was simply this: the pyro-acetic spirit acted in chronic bronchitis just as did other hydro-carbonates, and no better. The tar-water of Bishop Berkeley had a great reputation in its day, as had also the Barbadoes tar; the fetid gums belonged to the same class, and they all acted as stimulating expectorants to the bronchial membrane. In phthisis, when a cavity was present, much of the expectoration depended on the condition of the bronchial membrane; and hence the origin of the good effects of this vaunted remedy. To say it cured or arrested consumption was a mere delusion. All practitioners must have observed that any new remedy, from the confidence excited in the patient, did good for a time in chronic phthisis. Dr. Bird then showed the fallacy of the grounds on which naphtha had been established in temporary fame, and alluded to the singular circumstance, that its greatest, and certainly its most scientific, advocate, had died of consumption, his favorite remedy having been persevered in to the last.

Mr. Hird detailed the particulars of some cases of phthisis in which he had been induced to try the effects of the pyroacetic spirit. In no case did he see any benefit from its use. It must not be supposed, however, that the supporters of naptha employed this remedy solely; as extensive counter-irritation over the chest, iron, acetate of lead, and digitalis were employed at the same time, so as to make it difficult to determine which remedy really did good or harm.

Mr. A. Fisher related some cases of phthisis in which the remedy seemed to be of much service; but there appeared to be considerable obscurity in the diagnosis in these instances.

Dr. Willshire had given the remedy in a great number of cases of phthisis. It always did harm, and produced nausea and vertigo after having been administered for a period of a week or ten days. In one case of very doubtful character, it appeared to be of temporary service.—*Lancet.—Am. Jour. of Med. Science.*

